# Endothelial angiogenesis is directed by RUNX1T1-regulated VEGFA, BMP4 and TGF-β2 expression

**DOI:** 10.1371/journal.pone.0179758

**Published:** 2017-06-22

**Authors:** Ko-Hsun Liao, Shing-Jyh Chang, Hsin-Chuan Chang, Chen-Li Chien, Tse-Shun Huang, Te-Chia Feng, Wen-Wei Lin, Chuan-Chi Shih, Muh-Hwa Yang, Shung-Haur Yang, Chi-Hung Lin, Wei-Lun Hwang, Oscar K. Lee

**Affiliations:** 1 Institute of Microbiology and Immunology, National Yang-Ming University, Taipei, Taiwan; 2 Department of Obstetrics and Gynecology, Hsinchu Mackay Memorial Hospital, Hsinchu, Taiwan; 3 The Ph.D. Program for Translational Medicine, College of Medical Science and Technology, Taipei Medical University, Taipei, Taiwan; 4 Institute of Clinical Medicine, National Yang-Ming University, Taipei, Taiwan; 5 Immunity and Inflammation Research Center, National Yang-Ming University, Taipei, Taiwan; 6 Cancer Research Center, National Yang-Ming University, Taipei, Taiwan; 7 Division of Hematology-Oncology, Department of Medicine, Taipei Veterans General Hospital, Taipei, Taiwan; 8 Genomic Research Center, Academia Sinica, Taipei, Taiwan; 9 Department of Surgery, Taipei-Veterans General Hospital, Taipei, Taiwan; 10 School of Medicine, National Yang Ming University, Taipei, Taiwan; 11 Stem Cell Research Center, National Yang-Ming University, Taipei, Taiwan; 12 Department of Medical Research, Taipei Veterans General Hospital, Taipei, Taiwan; 13 Taipei City Hospital, Taipei, Taiwan; Medical College of Wisconsin, UNITED STATES

## Abstract

Tissue angiogenesis is intimately regulated during embryogenesis and postnatal development. Defected angiogenesis contributes to aberrant development and is the main complication associated with ischemia-related diseases. We previously identified the increased expression of RUNX1T1 in umbilical cord blood-derived endothelial colony-forming cells (ECFCs) by gene expression microarray. However, the biological relevance of RUNX1T1 in endothelial lineage is not defined clearly. Here, we demonstrate RUNX1T1 regulates the survival, motility and tube forming capability of ECFCs and EA.hy926 endothelial cells by loss-and gain-of function assays, respectively. Second, embryonic vasculatures and quantity of bone marrow-derived angiogenic progenitors are found to be reduced in the established *Runx1t1* heterozygous knockout mice. Finally, a central RUNX1T1-regulated signature is uncovered and VEGFA, BMP4 as well as TGF-β2 are demonstrated to mediate RUNX1T1-orchested angiogenic activities. Taken together, our results reveal that RUNX1T1 serves as a common angiogenic driver for vaculogenesis and functionality of endothelial lineage cells. Therefore, the discovery and application of pharmaceutical activators for RUNX1T1 will improve therapeutic efficacy toward ischemia by promoting neovascularization.

## Introduction

The controlled formation of blood vessels including vasculogenesis and angiogenesis is essential for embryonic development [1–3]. Embryonic vaculogenesis begins with endothelial differentiation from hemangioblasts and angioblasts in the presence of fibroblast growth factors (FGF) [4] followed by vascular endothelial growth factor (VEGF)-assisted assembly of primordial vessels [5]. The expression of BMP4 further enhances VEGF expression for outgrowth of an immature vascular system [6]. Angiogenesis occurs after vaculogenesis and acts through the recruitment of mesodermal progenitors and assembly of mesodermal precursors to pre-existing vessels for *de novo* blood vessel formation [7]. The expression of these angiogenic proteins are required for vasculogenesis-to-angiogenesis transition [8–10] and the homozygous deletion of VEGFA and BMP4 results in embryonic lethality [11–13].

Endothelial progenitor cells (EPCs) have been identified in mouse bone marrow [14], human peripheral blood human as well as cord blood [15, 16] for therapeutic angiogenesis. Upon tissue injuries, EPCs migrate to ischemic sites, proliferate and differentiate into endothelial cells (ECs) for regeneration [7, 17]. Human EPCs have been classified into two sub-populations; circulating angiogenic cells (CACs) and endothelial colony-forming cells (ECFCs) based on their phenotypic and functional properties [16, 18]. Specifically, ECFCs are able to generate tube-like structures, while CACs augment tubulogenesis by secreting paracrine factors including VEGFA [19, 20]. Poor angiogenesis and a lack of repair of the vasculature are main pathological features in diabetes, atherosclerosis and myocardial infarction [21–23]. The quantity of circulating ECFCs has been negatively correlated with the increased risk for cardiovascular disease [24, 25]. Mechanistically, the increased expression of miR-361-5p in diseased EPCs of coronary artery disease patients suppresses their angiogenesis capabilities by targeting VEGF expression [26].

RUNX1T1 (RUNX1 translocation partner 1), also named as ETO, CBFA2T1, MTG8 and ZMYND2, is a member of the conserved ETO family [27]. RUNX1T1 is originally identified through its involvement in a t(8;21) translocation associated with acute myeloid leukemia (AML) [28]. The AML-ETO fusion protein generated from a t(8;21) translocation is shown to disrupt normal hematopoiesis for leukemogenesis in both zebra fish and murine models [29, 30]. The expression of wild-type RUNX1T1 (ETO) is abundant in heart, brain and CD34 (+) progenitor cells [31, 32]. In addition, RUNX1T1 shows increased expression along with hematopoietic differentiation of embryonic stem cells [33]. In our previous study, the elevated expression of RUNX1T1 was observed in cord blood-derived ECFCs [19]. Given our rudimentary knowledge toward the functionality of wild-type RUNX1T1 (ETO) in endothelial cells and ECFCs, ECFCs were cultivated from cord blood and the Runx1t1 deficient mice were generated to interrogate impacts of RUNX1T1 on angiogenesis.

## Materials and methods

### Isolation and cultivation of primary endothelial cells and endothelial progenitor cells

The use of human materials, umbilical cords and cord blood, in this study was followed the Declaration of Helsinki and approved by the Institutional Review Board (IRB) of the MacKay Memorial Hospital, Hsinchu, Taiwan (IRB number: 12MMHIS025 and 15MMHIS200e). Female donors aged from 20 to 40 year old without significant disease and receiving any medication were enrolled and informed in accordance with certificated protocols. The written consents were obtained for all donors prior to the collect of umbilical cords and cord blood.

For isolation of primary human umbilical vein endothelial cells (HUVECs), fresh umbilical cords were obtained from donors. The isolated cells were cultured in M199 medium supplemented with heparin (20 U/ml, H3149, Sigma, St. Louis, USA), endothelial cell growth supplement (Millipore, Darmstadt, Germany), 10% fetal bovine serum (FBS) and 1% penicillin/streptomycin in flasks pre-coated with fibronectin (2.5 μg/cm^2^, Millipore). The culture medium was replaced by EGM2 with endothelial supplementation (Lonza Ltd., Basel, Switzerland) and 2% FBS at 6 hours after initial seeding and the medium was replenished every two days. The endothelial cell line EA.hy-926 cells (926-ECs) were purchased from ATCC and cultured in 10% FBS-containing DMEM medium in dishes pre-coated with fibronectin (Millipore).

For the isolation of endothelial precursor cells (EPCs), cord blood mononuclear cells (MNCs) were enriched by using LymphoprepTM (1.077 g/ml, StemCell, Vancouver, Canada). A total of 1 × 10^7^ MNCs were suspended in EGM2 containing endothelial supplements and 20% FBS, and placed on fibronectin-coated well. After four days of the initial seeding, the attached CACs appeared and the non-adherent cells were discarded. ECFCs emerged at 2–4 weeks after initial seeding and were cultivated under 2% FBS-containing EGM2. All the cells were trypsinized to prepare single-cell suspensions for further experiments. The first cell passage of ECFC (P1) was defined as the first appearance of ECFCs in culture. When ECFCs were confluent in a 10 cm dish, cells were then divided and defined as Pn+1. Primary cells with less than P7 were used for following experiments.

### Generation and characterization of the *Runx1t1* deficient mice

The animal study was approved by the Committee on the Ethics of Animal Experiments of the National Yang-Ming University (Permit No. 991245, 1000509 and 1060207) and conducted according to recommendation in the Institutional Animal Care and Use Committee of the National Yang-Ming University. All experimental mice were housed in a specific pathogen-free facility. The preparation of the targeting vector, selection of the targeted clones and the generation of the chimeric mice were conducted by the Gene Knockout Mouse Core Laboratory of National Taiwan University Center of Genomic Medicine. The *Runx1t1* conventional deletion mice were obtained using bacterial artificial chromosome clones derived from the 129J mouse strain; these were used to create the *Runx1t1* targeting vector by the recombineering method [34]. The targeting vector contained two *loxP* sites flanking exon 2 and exon 3 of *Runx1t1* and a neomycin selection cassette. The targeting vector was electroporated into 129J strain embryonic stem cells that had SOX2-driven cre expression [35]; followed by southern blot screening to identify the targeted clones. The target clones were injected into blastocysts of 129J/C57BL/6 mice and the chimeric mice were backcrossed to C57BL/6 male and female mice for changing the chromosome background. The *Runx1t1* null allele was transmitted for ten generations successfully. After ten generations, the C57BL/6 *Runx1t1* deficient mice were used for further investigation.

The PCR primer sequences were as follows: for retrieving the *Runx1t1* genomic DNA, these were AU2, BD2, YU2 and ZD2; for inserting the first *loxP* site and neo cassette, these were CU, DD2, EU2, and FD; for inserting the second *loxP* site, these were GU, HD2, IU2 and JD (listed in S1 Table). The targeting vector was then confirmed by sequencing. Following this, it was linearized by DNA digestion at the vector’s unique NotI and SpeI sites and electroporated into the ES cells. DNA from the tails of the genetically modified mice was harvested and used for genotyping by using the primer mixture (P1, P2 and P3 was labeled in green). All the primer sequences were listed in S1 Table. An amplicon of 388 base pairs was generated by using the P1 and P2 primers to examine the presence of the wild-type allele. An amplicon of 532 base pairs containing the *loxP* sequence was generated using the P1 and P3 primers and to validate the presence of the target allele. To characterize the *Runx1t1* deficient mice, the mice were scarified by CO_2_ asphyxiation to collect embryos, bloods, and organs for further investigation. The gross structures were inspected by using a dissecting microscope (Leica Microsystems, Wetzlar, Germany). Complete blood cell counts were measured by using an automated hematology analyzer (Sysmex, Taiwan) according to the manufacture’s procedures. For investigating thickness of vessel walls, aortas were proceed for hematoxylin and eosin stain (HE stain) and vessel thickness was measured every 90 degrees on captured images. Image analyses were performed by ImageJ software (National Institutes of Health, USA).

### Tube formation and transwell migration assay

For the tube formation assay, growth factor-reduced Matrigel (BD, 354230, Franklin Lakes, New Jersey, USA) was spread into the wells of a 96-well plate at 37°C for 1 hour to form a reconstituted basement membrane. 2x10^4^ cells were suspended in 100 μl of FBS containing medium (2% FBS containing EGM-2 medium for the ECFCs and HUVEC and 10% FBS containing DMEM was for EA.hy-926) and then seeded onto a Matrigel membrane for 6 hours at 37°C. The vascular structures formed were inspected by using an inverted light microscope (100X, Olympus, Tokyo, Japan). Images from five random fields per well were captured and the tube length in each image was calculated by MetaMorph Microscopy Automation and Image Analysis Software (Molecular Devices, Sunnyvale, CA, USA). For the transwell cell migration assay, 5x10^4^ cells were suspended in 100 μl of corresponding culture medium as described for the tube formation assay and introduced into the upper chamber of a transwell device with a membrane of 8.0 μm pores (Corning, 3422, NY, USA). 600 μl of 10% FBS-containing medium was added to the lower chamber to create a serum gradient. After 4 hours, the migrated cells were fixed and stained as described previously [26]. The migrated cells from five random fields per experimental condition were captured for quantification.

### *In vivo* Matrigel plug assay and hemoglobin assay

4x10^5^ cells were suspended in 150 ul EGM2 medium and mixed with an equal volume of growth factor-reduced Matrigel (354230, BD, New Jersey, USA) for subcutaneous injection in nude mice. The mice were then sacrificed at day 14 and the Matrigel plugs were extracted. The vasculature generated in the Matrigel plaque was inspected by using a dissecting microscope (Leica Microsystems, Wetzlar, Germany). For the hemoglobin assay, the Matrigel plug was immersed in RBC lysis buffer (150mM ammonium chloride (NH_4_Cl), 10mM potassium bicarbonate (KHCO_3_) and 0.12mM EDTA) to release the hemoglobin. After centrifugation, the supernatant was collected and mixed with 400 μl of Drabkin’s reagent (Sigma-Aldrich, USA) for 15 minutes at room temperature followed by optical detection at 540nm by using a multi-well scanning spectrophotometer (Thermo, Multiskan Spectrum).

### *In vivo* vessel permeability assay

The vessel permeability assay was performed as described previously [36]. Evans blue solution (0.5% Evans blue (Sigma-Aldrich, St. Louis, USA) in PBS) was prepared and filtered through 0.22 μm filter. The Evans blue solution was advanced into the lateral tail vein towards the direction of the head in mice for 60 minutes. The liver and heart were collected and immersed in 4% paraformaldehyde for 24 hours at 55°C before analyzing the absorbance of Evans blue (610 nm) by using a multi-well scanning spectrophotometer (Thermo, Multiskan, Spectrum).

### Plasmids, constructs and lentivirus production

pLenti4-Flag-*RUNX1T1* was constructed by cloning the coding sequences of RUNX1T1 into the RSRII site of pLenti4-Flag-CPO, which was kindly provided by Dr. Hsei-wei Wang, Institute of Microbiology and Immunology, National Yang-Ming University. The *RUNX1T1* shRNA (TRCN0000271292) and the control vector (pLKO_TRC005) were purchased from the RNAi Core Facility of the Academia Sinica, Taipei, Taiwan. All constructs were validated by direct sequencing. For lentivirus production, the target construct, VsVg, and △8.9 plasmids were co-transfected into 293T cells first and the lentivirus-containing supernatant was collected at 48 and 72 hours post initial transfection for further infection.

### Western blot analysis, flow cytometry and cell viability assay

Foe western blot, cells and tissues were harvested and lysed in protease inhibitor containing RIPA lysis buffers. After centrifugation for 15 minutes at 14000 × g (4°C), equal amounts of protein sample were separated on SDS-polyacrylamide gels and blotted onto PVDF membrane (Immobilon®, Millipore, USA). In a flow cytometry analysis, single cell suspension solution was prepared and cells were incubated with indicated antibodies for 1 hour. The flow cytometry analysis was performed by FACSAria Cell-Sorting System and FACSCalibur flow cytometer (BD Pharmingen, Franklin Lakes, NJ USA) with the assistance of Instrument Resource Cenrer (IRC), National Yang-Ming University. The results were further visualized by FlowJo (FlowJo LLC, Oregon, USA). All of the antibodies used in this study were listed in S2 Table. In cell viability assay, cells were incubated with 0.5 mg/mL MTT (Sigma-Aldrich, St. Louis, USA) for 2 hours at 37°C. The MTT solution was then discarded and 0.1% SDS prepared in 2-propanol were added for 5 minutes at room temperature to dissolve the purple formazan. The absorbance of end-point formazan was quantified by measuring absorbance at a wavelength of 570 nm by using a multi-well scanning spectrophotometer.

### RNA extraction and quantitative RT-PCR

Human cells and mice tissues were immersed in TRIzol® reagent (Life Technologies, Inc., Carlsbad, CA, USA) for the extraction of total RNA. All protocols followed manufacturer’s instructions with minor modifications. Total RNA ranging from 250 ng to 1 μg of total RNA was used to perform reverse transcription (RT) using the RevertAid™ Reverse transcriptase kit (Fermentas, Glen Burnie, Maryland, USA) according to manufacturer’s protocols. Real-time PCR reactions were performed using SensiFAST SYBR Hi-ROX mix (Bioline, Humber Road, London, UK), and the amplicons were detected and analyzed using the StepOne™ sequence detector (Life Technologies). The human gene expression was normalized to GAPDH expression. The mice gene expression level was normalized to Hprt1 expression. The relative expression was calculated by ΔΔCT methods.

### Gene expression and microarray analysis

Total RNA sample preparation, cRNA probe preparation, array hybridization, RT-qPCR validation and data analysis were carried out as described previously [37]. Default RMA settings were used to background correct, normalize and summarize all expression values via the R statistical programming language (http://www.r-project.org). In order to identify the RUNX1T1 regulated target genes in the ECFCs, the gene expression profiles of ECFC-pLKO and ECFC-sh *RUNX1T1* were analyzed by using Human Genome U133 Plus 2.0 chips (Affymetrix). The 5^th^ passage of ECFCs was used for transduction and microarray analysis. The core RUNX1T1 regulated signature was defined as the top 500 down-regulated genes (>1.5 folds) in ECFCs-sh*RUNX1T* cells described in S3 Table. Gene set enrichment analysis (GSEA) was then applied to assess the degree of association between the gene signature and various datasets obtained from the public domain [38]. All the down-regulated genes by RUNX1T1 (S3 Table) were subjected to explore the enriched biological activities by using Ingenuity Pathways Analysis (IPA) software (Ingenuity Systems, Redwood City, CA, USA). Genes regarding to the connective tissue development/ functioning, tissue development, organism development, organism survival and cardiovascular system development/ functioning in IPA knowledge database with *p*-values less than 0.001 were extracted for establishing connectivity network. The secretory and membranous proteins labeled in yellow were further defined as RUNX1T1-regulated secretome for validation. All the primer sequences for RT-qPCR are listed in S4 Table. The cDNA microarry datasets used for this study are listed as following. KSHV infected and non-infected endothelial cells (GSE16354). The gene expression microarray data for ECFC-pLKO and ECFC-sh*RUNX1T1* was deposited at Gene Expression Omnibus under the accession number GSE86594.

### Antibody neutralization

The neutralizing antibodies were added to cultured medium from RUNX1T1-overexpressed cells and treated EA.hy-926 cells (926 ECs) for 48 hours. The concentration of neutralizing antibodies against VEGFA, BMP4 and TGF-β2 was 4 μg/ml, 3 μg/ml and 1.25 μg/ml respectively. 4 μg/ml IgG antibody was used as a negative control.

### Statistical analysis

Independent sample t-tests and one-way ANOVA were performed to compare continuous variation between two groups. The Fisher’s exact test was applied for comparison of dichotomous variables. All of results are expressed as mean ± standard deviation (S.D.) of three or more biological replicates. *p*-values less than 0.05 were considered as significant. For animal studies, no statistical method was used to predetermine the sample size. The experiments were not randomised.

## Results

### Expression of RUNX1T1 positively correlates with the angiogenic activities of endothelial cells and endothelial precursor cells

**Fig 1:**
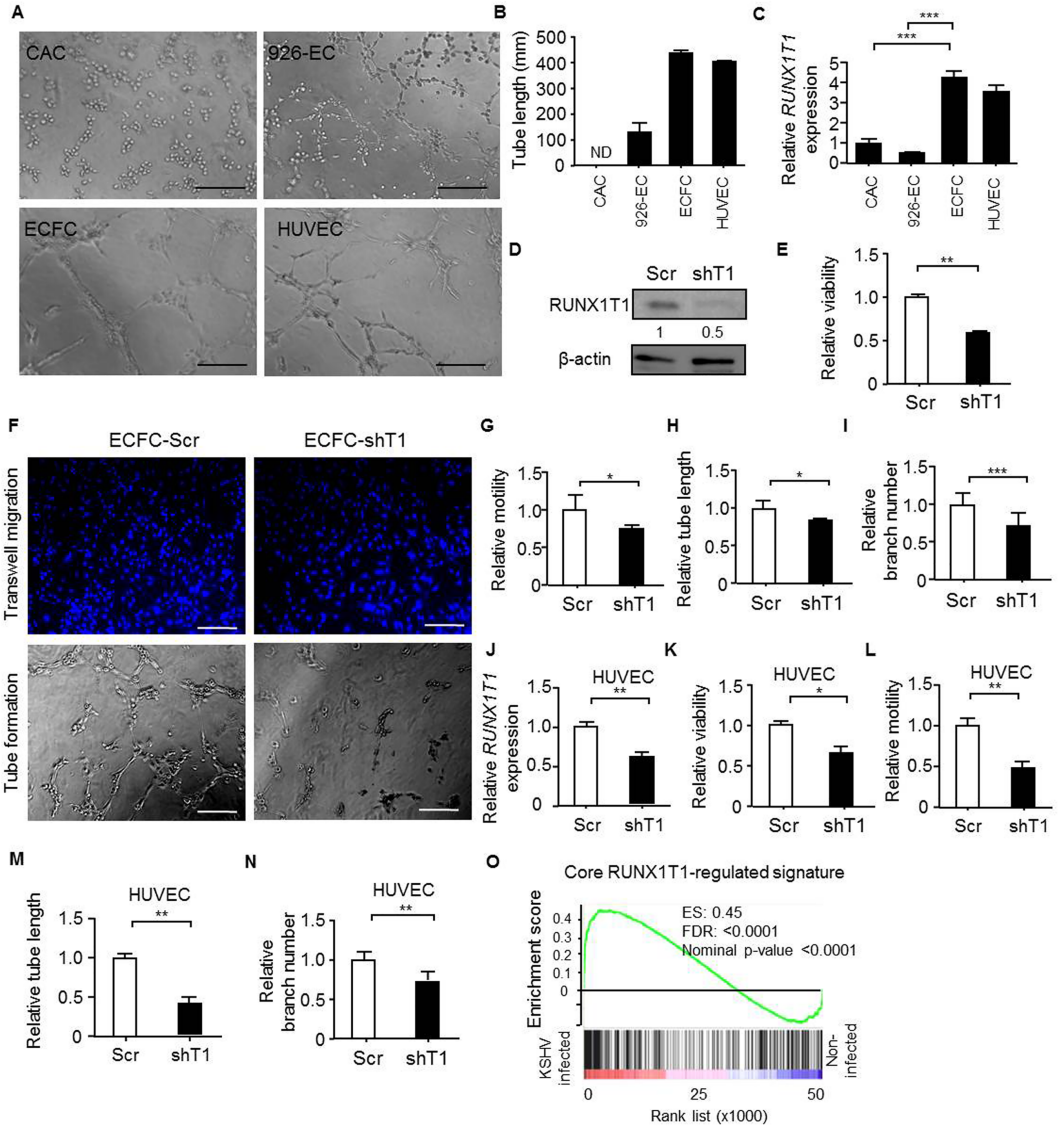
RUNX1T1 is required for angiogenic activity of ECFCs and HUVECs. (A) Representative pictures for illustrating the tube formation capacity of indicated cells. Scale bar = 50 μm (B) A histogram for showing endothelial tube lengths formed from the tube formation assay. N.D, none detected. Data represent mean± S.D. n = 3 independent experiments. (C) The RT-qPCR results for showing the relative expression levels of *RUNX1T1* at indicated cells. ***, *p*<0.001 (one-way ANOVA). (D) A Western blot showing the expression level of RUNX1T1. The band intensity was quantified and normalized to control cells. Scr, scramble control cells; shT1, *RUNX1T1-*knockdowned cells. (E) A histogram for showing the relative viability of ECFCs measured by MTT assay. **, *p*<0.01 (Student’s t test). (F) Representative images of the migration assay (upper panel) and the tube formation assay (lower panel). Scale bar = 50 μm. (G) A histogram for showing the relative motility of ECFCs. *, *p*<0.05 (Student’s t test). (H) A histogram showing the relative capillary formation ability of ECFCs. *, *p*<0.05 (Student’s t test). (I) A histogram for showing endothelial tube branch number from the tube formation assay. ***, *p*<0.001 (Student’s t test). (J) RT-qPCR results for showing the relative expression levels of *RUNX1T1* in HUVECs. **, *p*<0.001 (Student’s t test). (K) A histogram for showing the relative viability of HUVECs measured by MTT assay. *, *p*<0.05 (Student’s t test). (L). A histogram for showing the relative motility of HUVECs. **, *p*<0.01 (Student’s t test). (M). A histogram showing the relative capillary formation ability of HUVECs. **, *p*<0.01 (Student’s t test). (N). A histogram showing the relative capillary branch number of HUVECs. **, *p*<0.01 (Student’s t test). (O). The GSEA result showing a correlation between the core RUNX1T1 signature and the KSHV-infection signature (GSE16354). ES: enrichment score, FDR: false discovery rate.

**Fig 2:**
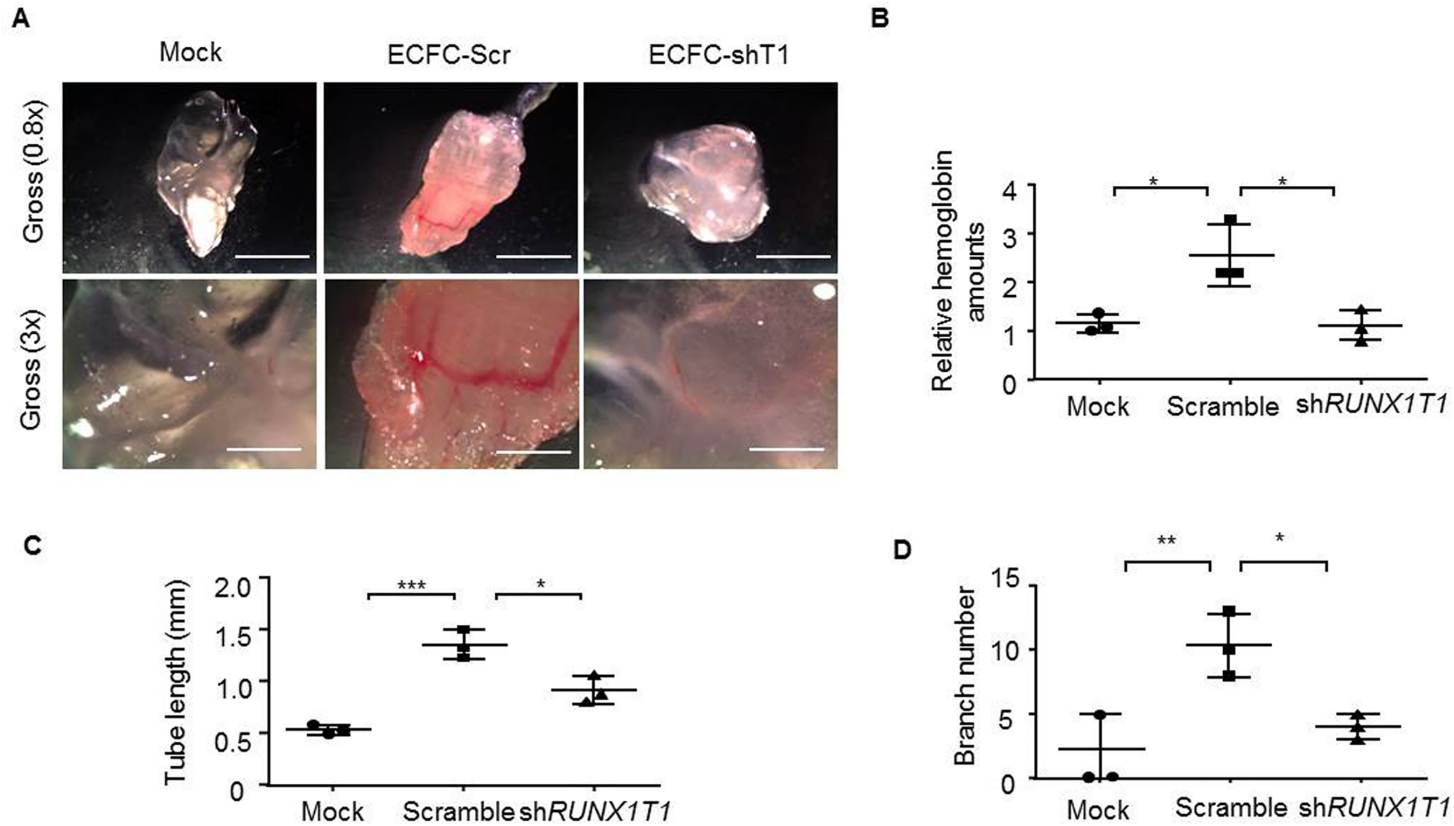
RUNX1T1 regulates vaculogenesis of ECFCs *in vivo*. (A) Representative images for *in vivo* Matrigel plug assay. n = 3. Scale bar = 3 mm (upper panel) and 1mm (lower panel). Arrows indicated vessels formed. (B) The relative amounts of hemoglobin extracted from the excised matrigel plugs. *, *p*<0.05 (one-way ANOVA) (C) A histogram showing the relative capillary formation ability in vivo matrigel plugs. *, *p*<0.05 (one-way ANOVA), ***, *p*<0.001 (Student’s t test). (D) A histogram showing the relative capillary branch number in vivo matrigel plugs. *, *p*<0.05 (one-way ANOVA), **, *p*<0.01 (Student’s t test).

In order to explore angiogenic effects of RUNX1T1 in EPCs and endothelial cells, we first compared the morphology of ECFCs and CACs from human umbilical cord blood and primary human umbilical vein endothelial cells (HUVECs). The isolated ECFCs showed cobblestone-like morphology (S1A Fig) and expressed progenitor cell marker CD34 as well as endothelial cell markers including CD31, KDR and VE-cadherin (S1B Fig), suggesting the maintenance of EPC features. It was shown that these expanded ECFCs exhibited longer tube length compared with that of CACs and 926-ECs (EA.hy-926 cells), an endothelial cell line commonly used for drug screening [39, 40] (Fig 1A and 1B). Next, it was found that the *RUNX1T1* expression pattern was positively associated with tube formation capacity (Fig 1C). To ascertain the RUNX1T1-mediated angiogenic effects, we transduced a shRNA against *RUNX1T1* or a scramble sequence into ECFCs and the expression of RUNX1T1 was confirmed by Western blotting (Fig 1D). It was found that knockdown of *RUNX1T1* in ECFCs attenuated cell viability (Fig 1E), reduced cell motility (Fig 1F, upper panel and Fig 1G) and limited the tube-forming capability (Fig 1F, lower panel and Fig 1H). The decreased RUNX1T1 expression down-regulated the tube branch numbers in primary ECFCs as well (Fig 1I). Similar results were reproduced in RUNX1T1 knock-downed HUVECs (Fig 1J–1N). RUNX1T1 is known as a transcription co-regulator during leukemogenesis [41] and may regulate angiogenic activities of endothelial lineage cells by modulating gene expression profile. It was found that the core RUNX1T1 signature consisted of the top 500 decreased genes (S3 Table) upon *RUNX1T1* knocking down was positively correlated with the gene expression profile of KSHV infected ECs known to exert enhanced angiogenic activity [42] by a gene set enrichment analysis (GSEA) (Fig 1O). Moreover, we also proved the importance of RUNX1T1 in vasculogenesis by *in vivo* Matrigel plaque assay. Matrigel vessels derived from *RUNX1T1* knock-downed ECFCs shown a reduced vascularity (Fig 2A) and lower levels hemoglobin amounts (Fig 2B). Knocking down *RUNX1T1* expression also reduced the tube formation lengths and branch number *in vivo* (Fig 2C and 2D).

**Fig 3:**
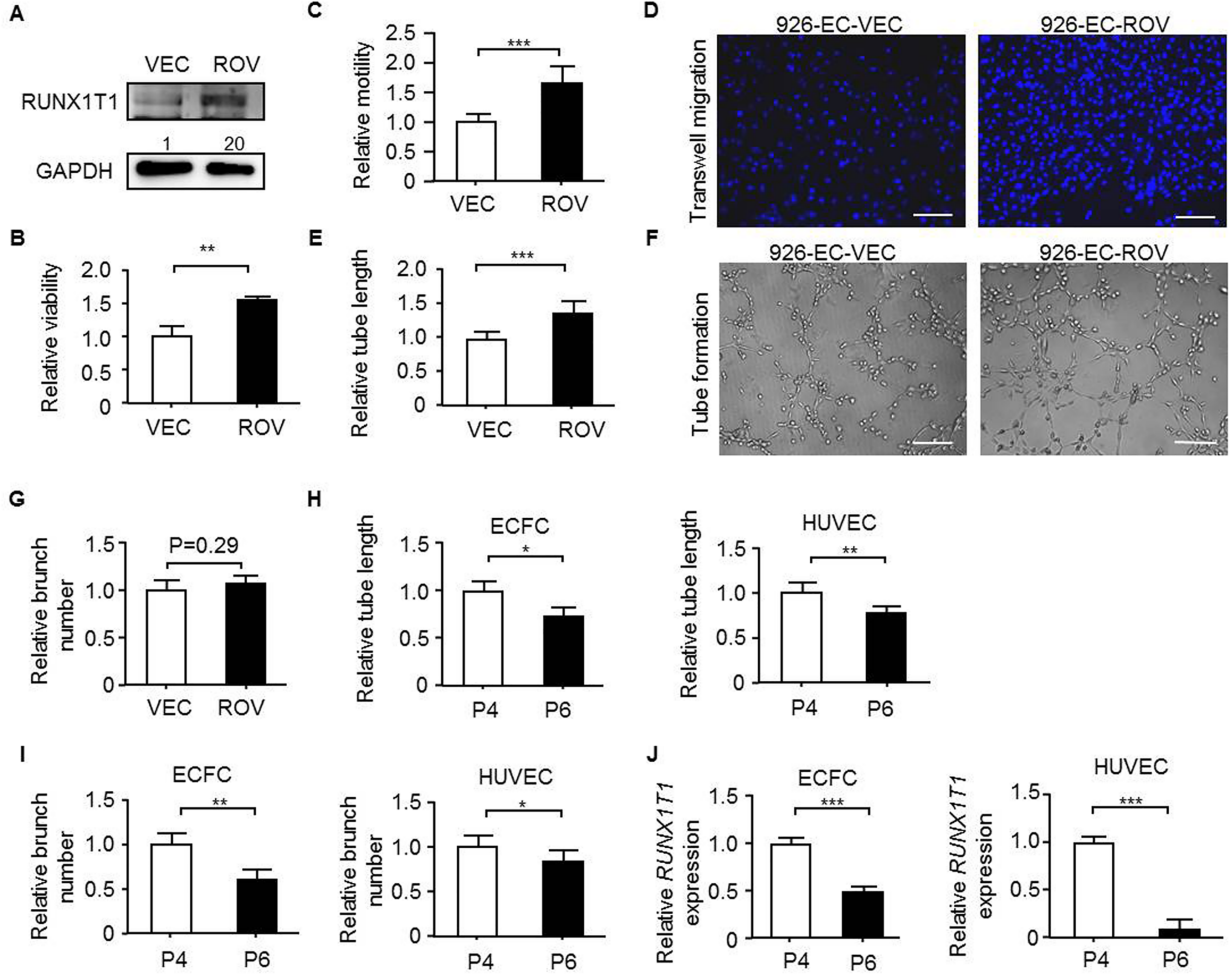
RUNX1T1 is sufficient to enhance angiogenic activity of endothelial cells. (A) Western blots showing for RUNX1T1 expression levels in 926-EC. VEC, vector control cells; ROV, ectopically *RUNX1T1*-expressing cells. The band intensity was quantified and normalized to control cells. (B) A histogram showing the relative viability of 926-EC measured by MTT assay. **, *p*<0.01 (Student’s t test). Data represent mean ± S.D. n = 3 independent experiments. (C) A histogram showing the relative motility of 926-EC. ***, *p*<0.001 (Student’s t test). (D) Representative images of transwell migration assays. Scale bar = 50 μm. (E) A histogram showing relative capillary formation ability of 926-EC. ***, *p*<0.001 (Student’s t test). (F) Representative images for the tube formation assays. Scale bar = 50 μm. (G) A histogram showing the relative capillary branch number of 926-ECs. (H) A histogram for showing the relative capillary formation capacity of the ECFCs (left panel) and the HUVECs (right panel) of different passage numbers. P4, the fourth passaged cells; P6, the sixth passaged cells. *, *p*<0.05; **, *p*<0.01 (Student’s t test). (I) A histogram for showing the relative capillary branch number of the ECFCs (left panel) and the HUVECs (right panel) of different passage numbers. *, *p*<0.05; **, *p*<0.01 (Student’s t test). (J) RT-qPCR results for showing the relative expression levels of *RUNX1T1* in ECFCs (left panel) and HUVECs (right panel) of different passage numbers. ***, *p*<0.001 (Student’s t test).

To investigate the RUNX1T1 function in another endothelial cells, we established a stable RUNX1T1-expressing cell line in 926-ECs (Fig 3A), which showed lower tube formation capacity (Fig 1B) and expressed a relatively low level of endogenous *RUNX1T1* (Fig 1C). It was found that forced expression of *RUNX1T1* in 926-ECs improved cell viability (Fig 3B), enhanced cell motility (Fig 3C and 3D) and increased the tube-forming capacity (Fig 3E and 3F) but not branch number (Fig 3G). It is reported that long-term cultivation of functional primary endothelial cells remains a challenge due to the loss of tube-forming capacity (Fig 3H) and decreased tube branch number (Fig 3I) along with cell passages [43]. A decreased expression of *RUNX1T1* was found to be proportional to the increment of cell passage number in both ECFCs and HUVECs (Fig 3J). Collectively, RUNX1T1 is required and essential for angiogenic activities both *in vitro* and *in vivo*.

### Knocking-out *Runx1t1* results in a decreased quantity of bone marrow-derived angiogenic progenitor cells and an impairment of vessel formation

**Fig 4:**
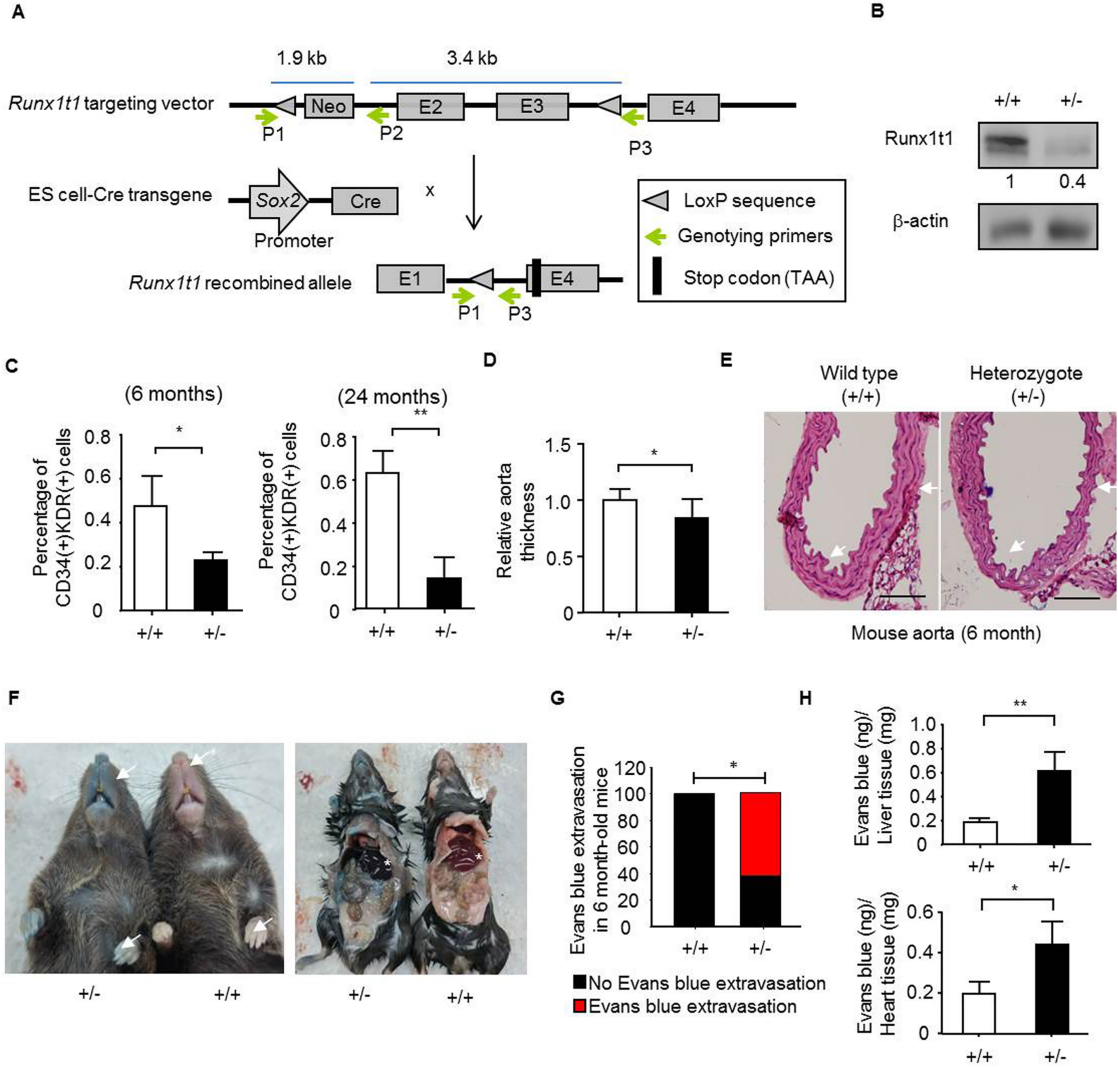
The decreased quantity of bone marrow-derived angiogenic progenitor cells and impaired aorta are observed in *Runx1t1* deficient mice. (A) A schematic representation of the gene-targeting strategy. The P1, P2 and P3 primers were used for the genotyping. The *loxP* sequence and a FRT-floxed neomycin cassette was introduced into intron 2 and another *loxP* sequence was inserted into intron 4 to allow Cre-ceconbinase-mediated removal of exons 2 and exon 3. E, exon; Kb, kilobase; Cre, cre-recombinase. (B) A Western blot showing the expression of Runx1t1 in the aorta of 6-month-old wild-type (+/+) and heterozygous *Runx1t1* knockout (+/-) mice. The band intensity was quantified and normalized to control cells. (C) The histograms for showing percentage of bone marrow-derived CD34 (+)/KDR (+) angiogenic precursor cells of indicated mice. *, *p*<0.05 (Student’s t-test); **, *p*<0.01 (Student’s t-test). Data represent mean ± S.D. n = 4 for each group (D) The quantification of thickness for aorta wall at indicated mice. n = 4 for each group, 6-month-old mice.*, *p*<0.05 (Student’s t test). (E) The histological images of aortas from indicated mice by hematoxylin and eosin staining. n = 4 for each group. Scale bar = 100 μm. (F). Representative pictures for Evans blue extravasation assay in the 6-month-old wild-type (+/+) and heterozygous *Runx1t1* knockout (+/-) mice. Arrows indicated relative location of extravasation observed in heterozygous *Runx1t1* knockout (+/-) mice. Star indicated the location of liver. (G) The histogram for showing percentage of aberrant vessel permeability in *Runx1t1* deficient mice. n (total mice number used for analysis) = 9 and 8 for 6-month-old wild-type mice (+/+) and *Runx1t1* heterozygous mice (+/-), respectively. *, *p*<0.05, (Fisher’s exact test). (H) The histogram for showing quantification of Evans blue extravasation in liver (upper panel) and heart (lower panel) of *Runx1t1* deficient mice (+/-).*, *p*<0.05; **, *p*<0.01 (Student’s t-test).

The vasculature is established during early development and required for normal physiological functions [44, 45]. To interrogate effects of RUNX1T1 on mammalian development, *Runx1t1* deficient mice were established. The mice gene modification was described in Fig 4A. The genotyping results of *Runx1t1* deficient mice showed that the target allele had been included in the F1 littermates (S2A Fig) and the decreased expression of Runx1t1 in the heterozygous mice was examined by Western blotting (Fig 4B), confirming the feasibility of this genetic tool. However, genotyping showed that live offspring of homozygous knock-out mice were not able to be obtained after weaning in a total of 157 offspring (S2B Fig). Therefore, heterozygous knock-out mice that were then used for further investigation. The bone marrows have been considered major sources for angiogenic cells. The bone marrow-derived CD34 (+)/ KDR (+) angiogenic progenitor cells are participated in ischemia regeneration [46] and bone marrow CD31 (+) cells represent the highly vasculogenic cells in bone marrow [47]. It was found that quantities of both CD34 (+)/ KDR (+) cells (Fig 4C) and CD31 (+) cells (S2C Fig) were reduced in the adult *Runx1t1* heterozygous knockout mice. By sorting CD31 (+) cells from 6-month-old *Runx1t1* heterozygous knockout mice, the decreased cell viability (S2D Fig) and motility (S2E Fig) were observed. Though the increased expression of RUNX1T1 has been reported in CD34 (+) hematopoietic progenitors [32], it was found that the red blood cells, white blood cells as well as platelet counts were not altered in *Runx1t1* heterozygous knockout mice (S2F Fig). The weights of organs and tissues examined including liver, kidney, spleen and white adipose tissue were not significantly changed in our *Runx1t1* heterozygous knockout mice (S2G Fig). On the contrary, it was found the aorta thickness was decreased in the heterozygous knockout mice (Fig 4D and 4E). As impaired vasculogenic cells and endothelial progenitor cells contribute to abnormal vessel structure [48], the extravasation of Evans blue dye were assessed [36]. It was found the *Runx1t1* heterozygous deficient mice showed increased vessel permeability (Fig 4F and 4G). Moreover, an increased Evans blue extravasation was observed in liver and heart tissue of *Runx1t1* heterozygous mice (Fig 4H). Collectively, Runx1t1 played an essential role in the maintenance of endothelial functions.

**Fig 5:**
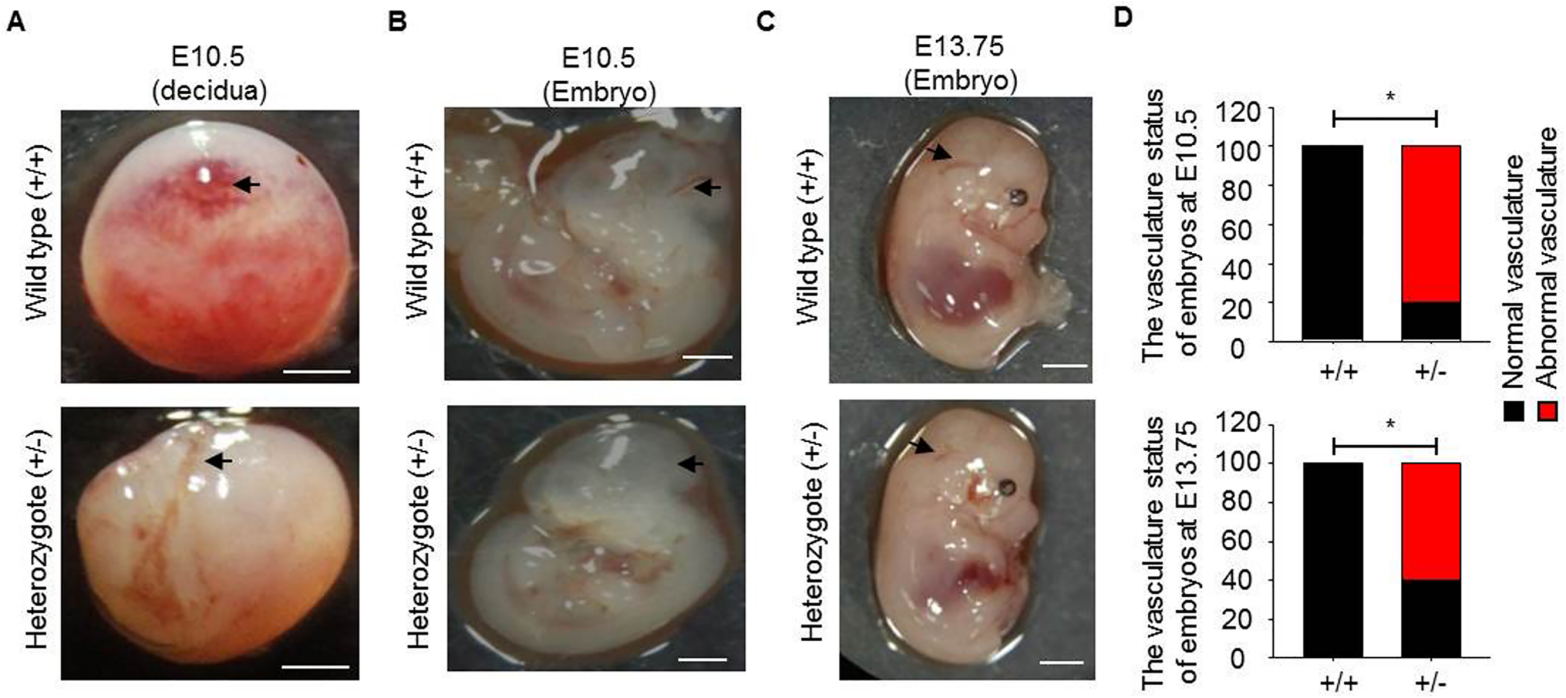
The aberrant vasculature formation is observed in *Runx1t1* deficient mouse embryos. (A) Images showing the vascularity of deciduas from a wild-type and a heterozygous *Runx1t1* knockout mouse at embryonic day 10.5 (E10.5). Scale bar = 1mm. Arrows indicated vessels formed. (B-C) Representative images showing vascularity of embryos from wild-type and heterozygous *Runx1t1* knockout mice at embryonic day 10.5 (B) and 13.75 (E13.75) (C) Scale bar = 1 mm. Arrows indicated cerebral vessel (CV) formed. (D) The histogram for showing percentage of aberrant vasculature in *Runx1t1* deficient embryos at E10.5 (upper panel) and E13.75 (lower panel). n (total mice number used for analysis) = 4, 5, 7, 5 for wild-type embryos (E10.5), *Runx1t1* deficient embryos (E10.5), wild-type embryos (E13.75) and *Runx1t1* deficient embryos (E13.75), respectively. *, *p*<0.05, (Fisher’s exact test).

In mouse embryos, endothelial cells are specified to form heart loop tubes and arch arteries from E7.5 to E10.5. The outflow tract and ventricles are formed at E13.5 [49]. To investigate on the effects of Runx1t1 on embryonic development, the mouse embryos from E10.5 to E13.75 were collected for characterization. The loss of live homozygous offspring was observed during E10.5 to E13.75 (S2H Fig) and indicated that homozygous deletion of *Runx1t1* resulted in embryonic lethality. The heterozygous decidua was found to have fewer vascular structures than that of the wild type mice at E10.5 (Fig 5A). These heterozygous embryos also exhibited impaired cerebral vessels at E10.5 (Fig 5B), E12.5 (S2I Fig) and E13.75 (Fig 5C) as pointed out by arrow. After calculation, we noted that more than sixty percent of heterozygous *Runx1t1* deficient embryos exhibited aberrant vasculatures (Fig 5D). Cumulatively, expression of RUNX1T1 is participated in vasculature development and maintenance of quantity of angiogenic progenitor cells.

### Identification of the central regulators involved in RUNX1T1-mediated angiogenic activity during blood vessel formation

**Fig 6:**
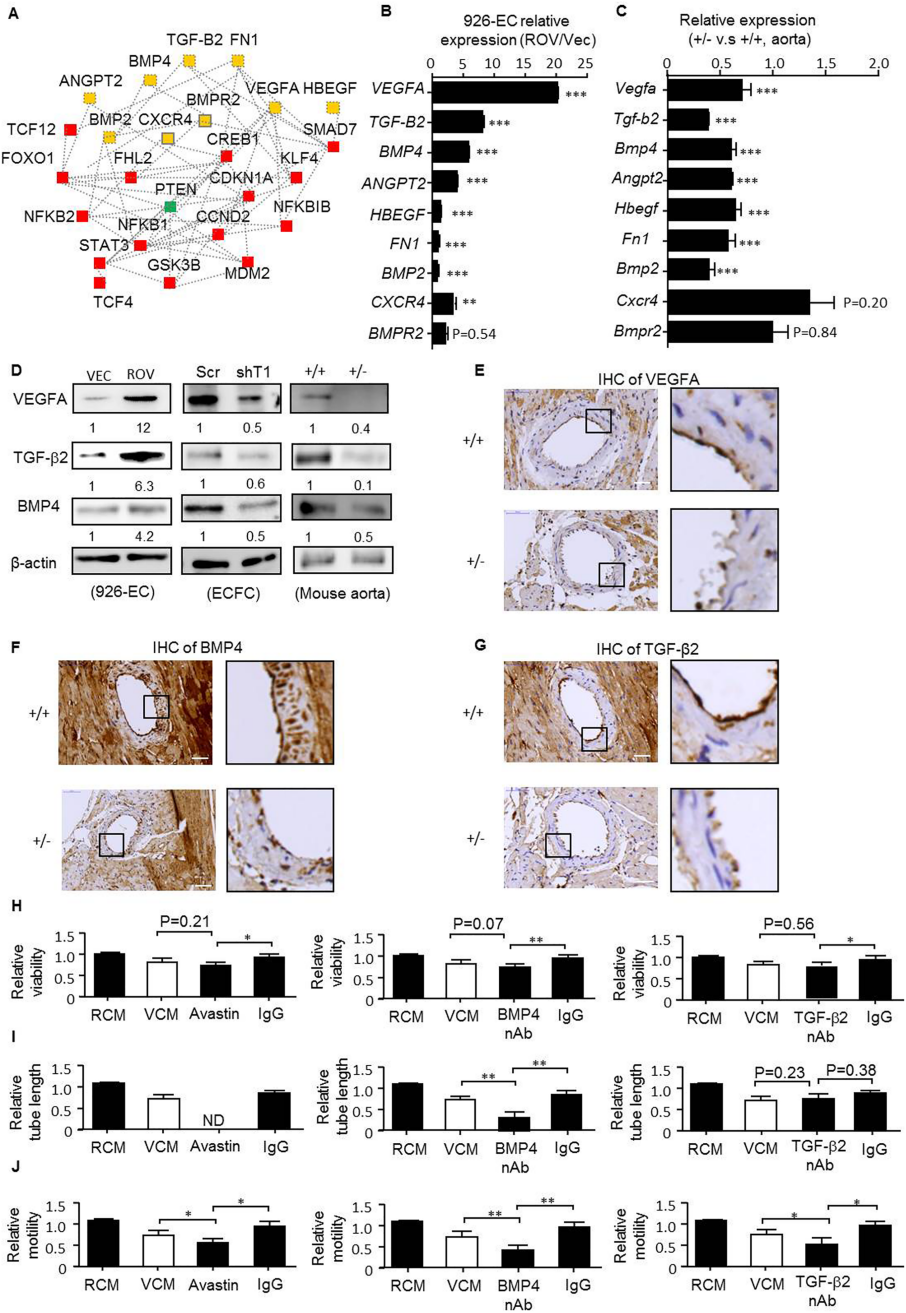
VEGFA, BMP4 and TGF-β2 mediate RUNX1T1-directed growth, motility and capillary-forming capacity in endothelial cells. (A) The connectivity network of RUNX1T1-regulated genes created by Ingenuity Pathway Analysis. Yellow, secreted or membranic proteins; Green, cytosolic proteins; Red, nuclear proteins. (B-C) RT-qPCR results for validating expression levels of selected genes marked in yellow in panel A in 926-ECs (B) and *Runx1t1* deficient mice (C) **, *p*<0.01; ***, *p*<0.001 (Student’s t test). Data represent mean ± S.D. n = 3 independent experiments. (D) Western blotting for showing the expression levels of VEGF-A, TGF-β2 and BMP4 at the indicated models. The band intensity was quantified and normalized to corresponding control cells. (E) The immunoreactivity of VEGFA of heart vessel. n = 2 for each group, 6-month-old mice. Scale bar = 50 μm. (F) The immunoreactivity of BMP4 of heart vessel. n = 2 for each group, 6-month-old mice. Scale bar = 50 μm. (G) The immunoreactivity of TGF-β2 of heart vessel. n = 2 for each group, 6-month-old mice. Scale bar = 50 μm. (H) A histogram for showing the relative viability at the indicated conditions (VEC-CM, the conditional medium from 926ECs-vector control cells; ROV-CM, the conditional medium from RUNX1T1-expressing 926ECs; nAb, neutralizing antibody). *, *p*<0.05 and **, *p*<0.01 (one-way ANOVA). (I) A histogram for showing the relative capillary formation ability at indicated conditions. N.D, none detected. **, *p*<0.01, (one-way ANOVA). (J) A histogram for showing the relative cell migration ability at indicated conditions. *, *p*<0.05 and **, *p*<0.01, (one-way ANOVA).

Given that gene functions work together to bring about biological processes and RUNX1T1 expression is highly involved in vasculogenesis and EPC development, we then investigated the central RUNX1T1 signature in this context. Genes related to a number of relevant categories, namely connective tissue development and function, tissue development, organism development, organism survival and cardiovascular system development with a *p-*value less than 0.001 were selected to establish a connetivity network by IPA (Fig 6A). Secreted and membranous proteins associated with angiogenesis were put forward for validation due to their druggable potential as shown in Fig 6B and 6C. The expression of VEGFA, TGF-β2, BMP4, Angiopoietin-2, HBEGF, Fibronectin 1, BMP2, CXCR4 and BMPR2 was increased in ectopically RUNX1T1-expressing 926-ECs (Fig 6B) and consistently, decreased expression of these genes were observed in *Runx1t1* heterozygous knockout mice compared to that of corresponding controls (Fig 6C). As the fold increment of VEGFA, TGF-β2 and BMP4 were evident upon overexpression of RUNX1T1 (Fig 6B), they were put forward as model target. First, the reduced expression of VEGFA, BMP4 and TGF-β2 were further confirmed by Western blotting in RUNX1T1 knock-downed ECFCs, ectopically RUNX1T1-expressing 926-ECs and heterozygous adult mice aorta as showed in Fig 6D. A decreased expression of VEGFA, BMP4 and TGF-β2 in heart endothelium of *Runx1t1* deficient mice were noted *in vivo* (Fig 6E–6G). Second, the inactivation of VEGFA, BMP4 and TGF-β2 signaling by neutralizing antibodies abolished the RUNX1T1-promoted cell viability (Fig 6H), tube forming capacity (Fig 6I) and cell motility (Fig 6J). Taken together, our results demonstrated that RUNX1T1 promoted endothelial angiogenic activities by activating the expression of VEGFA, BMP4 and TGF-β2.

## Discussion

The RUNX1T1 (ETO) is first identified as a fusion partner to AML1 (RUNX1) in leukemia [50]. The RUNX1-RUNX1T1 (AML-ETO), a pathological fusion protein containing the amino-terminal 177 amino acids of AML1 and the carboxyl-terminal 575 amino acids of RUNX1T1 is generated from a t(8;21) translocation that disrupts normal hematopoiesis [50]. It is reported RUNX1T1 acts as competing endogenous RNA for titrating micro RNAs in AML [51]. Given that the majority studies regarding functionality of RUNX1T1 (ETO) centered at AML-ETO fusion protein and pathogenesis for AML, molecular behaviors of wild-type RUNX1T1 in endothelial lineage cells remain elusive. In physiological condition, ETO suppresses activity of C/EBPβ during early adipogenesis [52]. The FTO regulated alternative splicing of RUNX1T1 further modulates preadipocyte differentiation [53] More RUNX1T1 is suggested to regulate pancreas development by regulating pancreatic polypeptide and Ghrelin expression [54]. In pathological condition, RUNX1T1 is shown to promote microglial proliferation by regulating CDK4 [55], but represses the proliferation and increases 5-flurouracil (5-FU) sensitivity in a colon cancer cell line (HCT116) [56]. These findings suggest pleiotropic effects of RUNX1T1 on different cells during development and disease progression.

In our study, lethality of *Runx1t1* deficient embryos was found to occur before E10.5 as no homozygotes were identified at E10.5 (S2H Fig), which coincided with lethality in VEGFA and BMP4 deficient embryos at E8.5 [57] and E9.5 [12], respectively, indicating VEGFA and BMP4 may be regulated by RUNX1T1 during early embryonic development. The expression of TGF-β2 in indispensable for proliferation of smooth muscle cells (SMCs) [58] and recruitment of SMCs to lymphatic vessels stimulates the secretion of endothelial-derived Reelin for lymphatic vessel morphogenesis [59]. Here, we identified VEGFA, BMP4 and TGF-β2 as RUNX1T1-regulated secretory proteins in human and their expression positively associated with RUNX1T1 both in vitro and in vivo (Fig 6B–6G). The molecular behaviors by which wild-type RUNX1T1 acted in endothelial lineage cells were further explored by neutralizing antibodies and our studies demonstrated that RUNX1T1 directed angiogenesis by activating a number of angiogenic factors at least in part, namely VEGFA, BMP4, and TGF-β2.

Lines of evidence have suggested impacts of RUNX1T1 in neurological development. First, the RUNX1T1 (MTG8) is belonged to the highly conserved MTG family [60] known to induced by proneural genes including ASCL1 during neurogenesis in mice [61]. Second, Drosophila homolog of ETO/MTG8, *nervy*, acts downstream of *achaete* (*ac*) and *scute* (*sc)* for mechanosensory organ development emanating from the sensory organ precursor (SOP) by reinforcing Delta-mediated Notch signaling [62]. Third, XETOR, a homolog of human oncoprotein ETO/MTG8 in Xenopus, is required for regulating the size of proneural domain [63] In our study, fainting and convulsive phenotypes were observed in 8% adult *Runx1t1* heterozygous mice (13/ 157) as shown in S1 Movie, implying functionality of RUNX1T1 in neurological behaviors. On the other hand, RUNX1T1 is demonstrated to regulate gastrointestinal development by insertional inactivation [64]. The postnatal viability was greatly reduced and the aberrant gut architecture as well as rectal hemorrhage was observed in established homozygous mice (MGI2660657). In our *Runx1t1* heterozygous mice, evident extravasation of Evan Blue dye in intestinal track was noted. The phenotype variation may result from mice genetic background effect.

Currently, we demonstrated clearly that VEGFA, BMP4 and TGF-β2 mediated RUNX1T1-directed angiogenesis in endothelial cells. To the best of our knowledge, this is the first evidence that demonstrates wild-type RUNX1T1 is involved in endothelial angiogenesis. The gene regulation mode exerted by RUNX1T1 has been suggested to be epigenetic regulation through a protein-protein interaction [41, 65]. Therefore, identification of transcription regulators bound with RUNX1T1 for eliciting RUNX1T1-orchestrated secretome in endothelial lineage cell is intriguing but undecided. Also, effects of RUNX1T1 signaling in endothelium smooth muscle cells interplay remain to be elucidated. Eventually, the discovery of pharmaceutical RUNX1T1 activator will shed slight on therapeutic angiogenesis toward vascular diseases.

## Supporting information

S1 Fig The characterization of ECFCs expanded from human cord bloods. (A) Representative pictures for illustrating the morphology of ECFCs and HUVECs. Scale bar = 50 μm (B) Flow cytometry results for showing the progenitor marker (CD34) and endothelial markers (CD31, KRD, VE-cadherin) in ECFCs and HUVECs.
(TIF)

S2 Fig The characterization of *Runx1t1* deficient mice. (A) Representative images for showing genotyping results for four littermates at 3–4 week-old F1 generation. Amplicons of the wild-type and targeted alleles are 388 and 532 base pairs in length, respectively. M, DNA ladder; bp, base pairs. (B) A table for summarizing the breeding program for the 3–4 week-old adult mice. (C) A histogram for showing percentage of bone marrow-derived CD31 (+) vasculogenic cells of indicated mice n = 4 for each group, 24-month-old mice. *, *p*<0.05 (Student’s t-test). (D) A histogram for showing the relative viability of CD31 (+) vasculogenic cells measured by MTT assay. n = 3 for each group, 6-month-old mice. ***, *p*<0.001 (Student’s t test). (E) A histogram for showing the relative motility of CD31 (+) vasculogenic cells. n = 3 for each group, 6-month-old mice. **, *p*<0.01 (Student’s t test). (F) A scatter plot showing the RBC count (upper panel), WBC count (middle panel) and. platelet count (lower panel) (*p*-value is estimated by Student’s t test). Data represent mean ± S.D. RBC, red blood cell, WBC, white blood cell. (G). A table for summarizing weights of selected organs and tissues at 22–26-week-old adult mice. (H). A table summarizing the breeding program at the embryonic day 10.5–13.75. (I). Representative images showing the vascularity of embryos from a wild-type and heterozygous *Runx1t1* knockout mouse at embryonic day 12.5 (E12.5). Scale bar = 1 mm. Black arrows indicated cerebral vessel (CV) formed.
(TIF)

S1 Table The primer sequences for recombination and genotyping. (XLS)

S2 Table The antibody list. (XLS)

S3 Table The down-regulated genes in shRUNX1T1-ECFC cells compared with vector control cells (1.5 folds). (XLS)

S4 Table The primers for RT-qPCR. (XLS)

S5 Table The Ct value of qPCR results. (XLS)

S1 Movie The neurological abnormality in *Runx1t1* heterozygous mice. (MP4)
