# Supplementary figures and images for: Endothelial angiogenesis is directed by RUNX1T1-regulated VEGFA, BMP4 and TGF-β2 expression
Source: PLoS One. 2017 Jun 22;12(6):e0179758. doi: 10.1371/journal.pone.0179758 (PMC5481149; doi:10.1371/journal.pone.0179758)

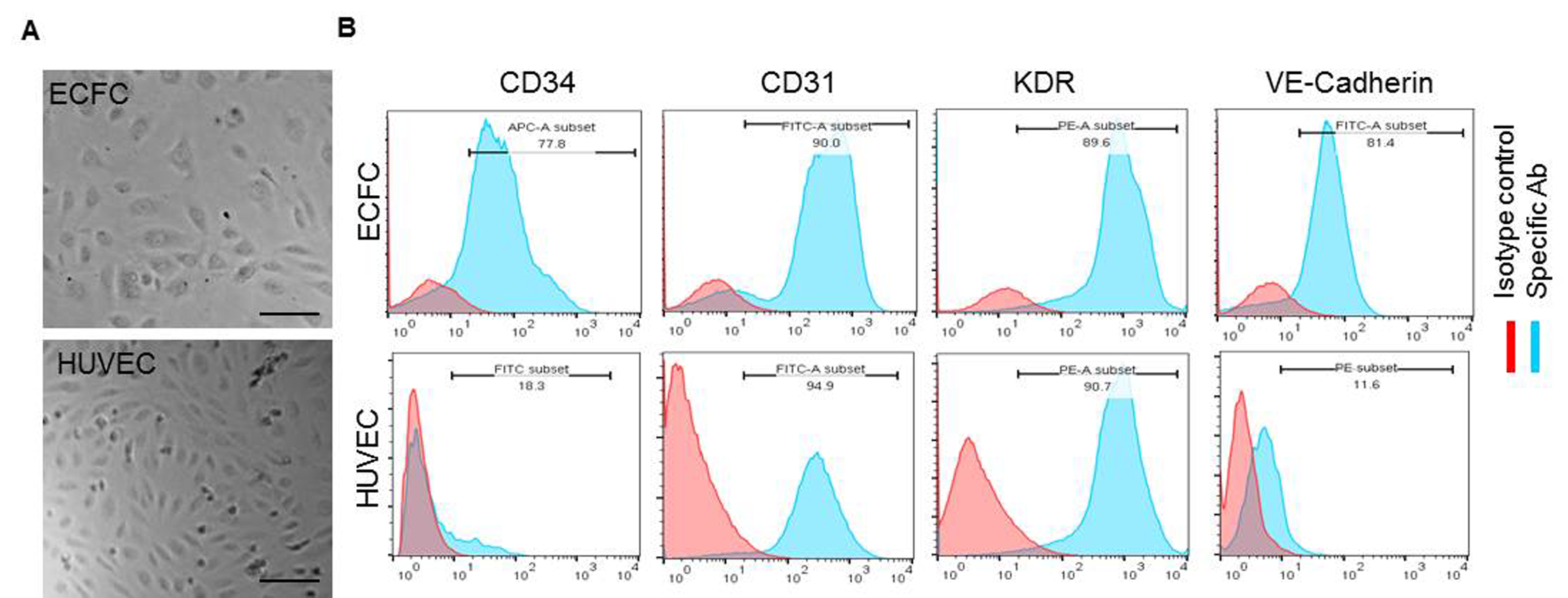

Supplement: S1 Fig — (A) Representative pictures for illustrating the morphology of ECFCs and HUVECs. Scale bar = 50 μm (B) Flow cytometry results for showing the progenitor marker (CD34) and endothelial markers (CD31, KRD, VE-cadherin) in ECFCs and HUVECs. (TIF) [file pone.0179758.s001.tif]

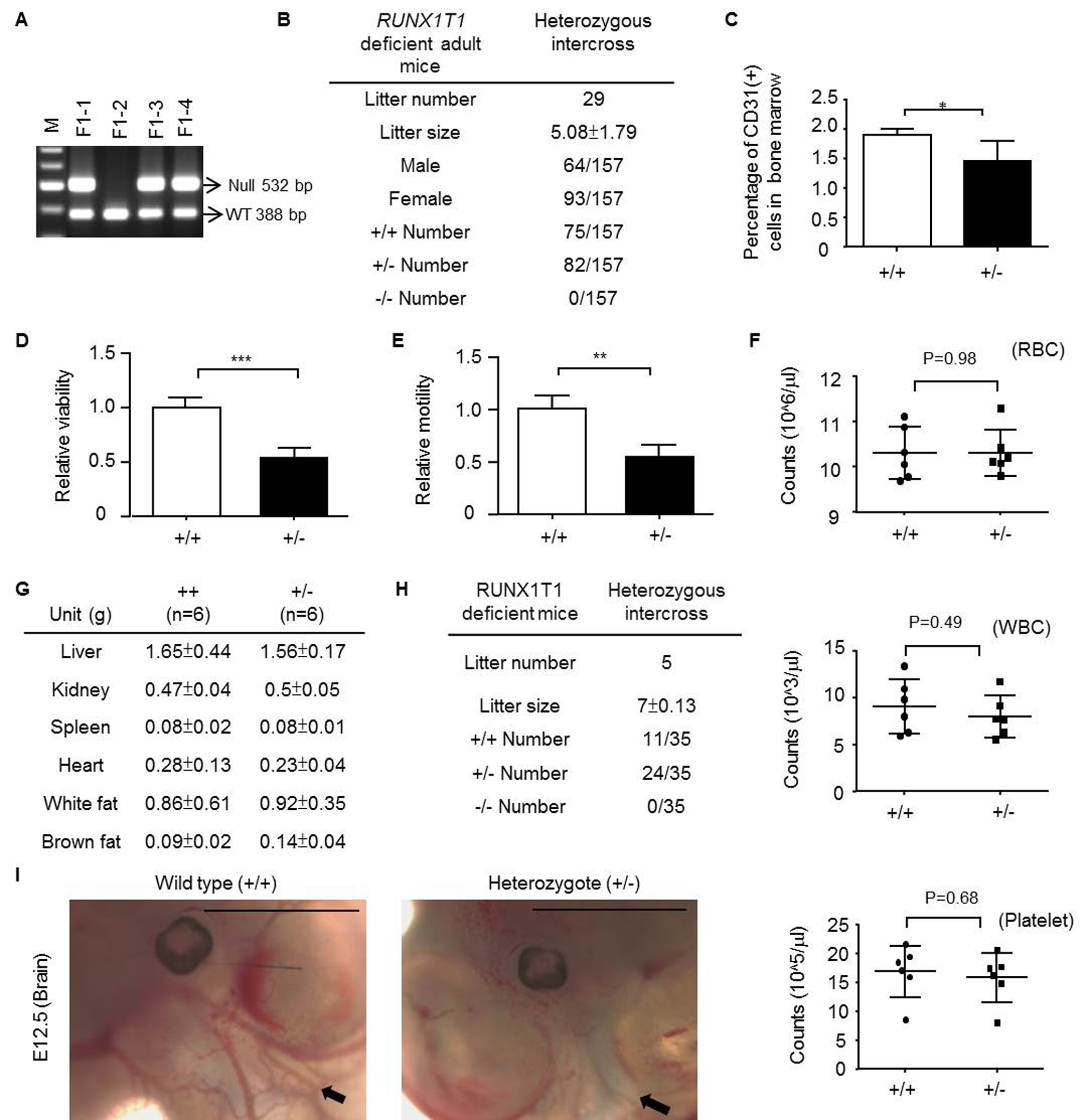

Supplement: S2 Fig — (A) Representative images for showing genotyping results for four littermates at 3–4 week-old F1 generation. Amplicons of the wild-type and targeted alleles are 388 and 532 base pairs in length, respectively. M, DNA ladder; bp, base pairs. (B) A table for summarizing the breeding program for the 3–4 week-old adult mice. (C) A histogram for showing percentage of bone marrow-derived CD31 (+) vasculogenic cells of indicated mice n = 4 for each group, 24-month-old mice. *, p<0.05 (Student’s t-test). (D) A histogram for showing the relative viability of CD31 (+) vasculogenic cells measured by MTT assay. n = 3 for each group, 6-month-old mice. ***, p<0.001 (Student’s t test). (E) A histogram for showing the relative motility of CD31 (+) vasculogenic cells. n = 3 for each group, 6-month-old mice. **, p<0.01 (Student’s t test). (F) A scatter plot showing the RBC count (upper panel), WBC count (middle panel) and. platelet count (lower panel) (p-value is estimated by Student’s t test). Data represent mean ± S.D. RBC, red blood cell, WBC, white blood cell. (G). A table for summarizing weights of selected organs and tissues at 22–26-week-old adult mice. (H). A table summarizing the breeding program at the embryonic day 10.5–13.75. (I). Representative images showing the vascularity of embryos from a wild-type and heterozygous Runx1t1 knockout mouse at embryonic day 12.5 (E12.5). Scale bar = 1 mm. Black arrows indicated cerebral vessel (CV) formed. (TIF) [file pone.0179758.s002.tif]
